# Effects of Tryptophan and Physical Exercise on the Modulation of Mechanical Hypersensitivity in a Fibromyalgia-like Model in Female Rats

**DOI:** 10.3390/cells13191647

**Published:** 2024-10-03

**Authors:** Rafael Marins Rezende, Roney Santos Coimbra, Markus Kohlhoff, Lukiya Silva Campos Favarato, Hércia Stampini Duarte Martino, Luciano Bernardes Leite, Leoncio Lopes Soares, Samuel Encarnação, Pedro Forte, António Miguel de Barros Monteiro, Maria do Carmo Gouveia Peluzio, Antônio José Natali

**Affiliations:** 1 Department of Physiotherapy, Universidade Federal de Juiz de Fora, Governador Valadares 35020-360, MG, Brazil; faelfisio@yahoo.com.br; 2 Instituto Rene Rachou–Fiocurz Minas, Belo Horizonte 30190-009, MG, Brazil; roney.coimbra@fiocruz.br (R.S.C.); markus.kohlhoff@fiocruz.br (M.K.); 3 Department of Veterinary Medicine, Universidade Federal de Viçosa, Viçosa 36570-900, MG, Brazil; lscampos@ufv.br; 4 Department of Nutrition and Health, Universidade Federal de Viçosa, Viçosa 36570-900, MG, Brazil; hercia@ufv.br (H.S.D.M.); mpeluzio@ufv.br (M.d.C.G.P.); 5 Exercise Biology Laboratory, Department of Physical Education, Universidade Federal de Viçosa, Viçosa 36570-900, MG, Brazil; luciano.leite@ufv.br (L.B.L.); leoncio.lopes@ufv.br (L.L.S.); 6 Department of Sports Sciences, Instituto Politécnico de Bragança, 5300-253 Bragança, Portugal; pedromiguelforte@gmail.com (P.F.); mmonteiro@ipb.pt (A.M.d.B.M.); 7 Instituto Politécnico de Bragança, Campus de Santa Apolónia, 5300-253 Bragança, Portugal; samuel01.encarnacao@gmail.com; 8 Department of Physical Education, Sport and Human Movement, Universidad Autónoma de Madrid, 28049 Madrid, Spain; 9 Research Centre for Active Living and Wellbeing (Livewell), Instituto Politécnico de Bragança, 5300-253 Bragança, Portugal; 10 CI-ISCE, Instituto Superior de Ciências Educativas do Douro (ISCE Douro), 4560-547 Penafiel, Portugal; 11 Research Centre in Sports Sciences, Health Sciences and Human Development, 5001-801 Vila Real, Portugal

**Keywords:** fibromyalgia, treadmill running, brain, hypersensitivity, tryptophan, serotonin, kynurenine, indolamine

## Abstract

Though the mechanisms are not fully understood, tryptophan (Trp) and physical exercise seem to regulate mechanical hypersensitivity in fibromyalgia. Here, we tested the impact of Trp supplementation and continuous low-intensity aerobic exercise on the modulation of mechanical hypersensitivity in a fibromyalgia-like model induced by acid saline in female rats. Twelve-month-old female Wistar rats were randomly divided into groups: [control (n = 6); acid saline (n = 6); acid saline + exercise (n = 6); acid saline + Trp (n = 6); and acid saline + exercise + Trp (n = 6)]. Hypersensitivity was caused using two intramuscular jabs of acid saline (20 μL; pH 4.0; right gastrocnemius), 3 days apart. The tryptophan-supplemented diet contained 7.6 g/hg of Trp. The three-week exercise consisted of progressive (30–45 min) treadmill running at 50 to 60% intensity, five times (Monday to Friday) per week. We found that acid saline induced contralateral mechanical hypersensitivity without changing the levels of Trp, serotonin (5-HT), and kynurenine (KYN) in the brain. Hypersensitivity was reduced by exercise (~150%), Trp (~67%), and its combination (~160%). The Trp supplementation increased the levels of Trp and KYN in the brain, and the activity of indoleamine 2,3-dioxygenase (IDO), and decreased the ratio 5-HT:KYN. Exercise did not impact the assessed metabolites. Combining the treatments reduced neither hypersensitivity nor the levels of serotonin and Trp in the brain. In conclusion, mechanical hypersensitivity induced by acid saline in a fibromyalgia-like model in female rats is modulated by Trp supplementation, which increases IDO activity and leads to improved Trp metabolism via the KYN pathway. In contrast, physical exercise does not affect mechanical hypersensitivity through brain Trp metabolism via either the KYN or serotonin pathways. Because this is a short study, generalizing its findings warrants caution.

## 1. Introduction

A syndrome of persistent and diffuse pain defines fibromyalgia. Two to five percent of the population experience such discomfort, especially middle-aged and old women [1,2]. Fibromyalgia is connected to deficits in the endogenous systems that modulate pain [3,4,5]. For instance, because efferent serotoninergic neurons negatively modulate the levels of substance P, low and high levels of serotonin and substance P, respectively, in the fluid in the brain ventricles and surrounding the spinal cord of individuals with fibromyalgia indicate modifications in the central neurotransmissions, thus resulting in alterations in the central response to pain [6,7,8,9].

Since tryptophan (Trp) is an originator of serotonin, disruptions in Trp metabolism appear to play an important part in the etiology of fibromyalgia. Trp metabolism has two main pathways that result in two different metabolites: serotonin (5-HT) and kynurenine (KYN). While the breakdown of 5-HT produces 5-hidroxiindolacetic acid (5-HIAA), KYN is further converted into neuroactive metabolites (i.e., quinolinic acid) [10]. An inverse relationship between the brain levels of Trp, 5-HT, 5-HIAA, and clinical pain measures and a positive interrelationship between the levels of KYN and the perception of pain are suggested [11,12,13]. 

An animal model of acid saline-induced fibromyalgia has been used to evaluate prophylactic and curative therapies and to investigate its underlying mechanisms. For example, diet and physical exercise have been tested as adjuvant non-pharmacological strategies to care for fibromyalgia. Concerning diet, a diet supplemented with Trp has been tested in acid saline-induced fibromyalgia to augment its availability in the brain, which would boost the modulatory actions of the serotoninergic system in pain control [14,15,16,17,18]. Supplementation with Trp thus appears to improve serotonin function in the brain, which reduces cortisol secretion during situations of high stress [19]. Regarding physical exercise, previous studies demonstrated that animals with acid saline-induced fibromyalgia had their mechanical hyperalgesia attenuated after performing aerobic exercise regimes [20,21,22]. It appears that aerobic exercise activates endogenous inhibitory mechanisms, including opioids and serotonin, and reduces the systemic levels of biomarkers for anxiety (e.g., cortisol, norepinephrine) and infection (e.g., cytokines). 

Despite such evidence, the efficiency of supplementation with Trp and aerobic exercise on the modulation of mechanical hypersensitivity is not well understood and requires further investigation. Thus, we evaluate the impact of a Trp-supplemented diet and continuous low-intensity aerobic exercise on modulating mechanical hypersensitivity in a fibromyalgia-like model in female rats. 

## 2. Materials and Methods

### 2.1. Animals and Study Design

Because fibromyalgia is experienced mainly by middle-aged and old women [1,2], female Wistar rats at 12 months of age were assigned to one of the following groups: C (control, n = 6); AS (acid saline, n = 6); ASE (acid saline + exercise, n = 6); ASTrp (acid saline + Trp, n = 6); and ASETrp (acid saline + exercise + Trp, n = 6). Throughout experiments (21 days), rats were housed individually in cages in the animal house (22 ± 2 °C), with a 12/12 h light/dark cycle and water and food ad libitum (see below). One day after the acid saline injection, exercised rats underwent a continuous aerobic exercise program (see below). All procedures in the experiments followed the National Guidance for the Care and Use of Laboratory Animals and had the consent of the Institutional Ethics Committee (CEUA-UFV 21/2015).

### 2.2. Induction of Hypersensitivity

Hypersensitivity was induced as described by Sluka et al. [23]. The rats were kept in sedation using isoflurane, 100% FiO2, with instinctive ventilation. Rats in the AS groups were injected two doses of acid saline (20 μL; pH 4.0) intramuscularly in the right hind limb (gastrocnemius) 3 days apart. The rats used as controls were injected with the same dose of saline (pH 7.4).

### 2.3. Dietary Supplementation

Rats were given water and food ad libitum over the experiment (21 days). Purified diets (i.e., control and supplemented) were pelletized (RHOSTER, São Paulo, Brazil). Food ingestion and efficiency and weight gain were determined. Diet composition followed the AIN-93M (American Institute of Nutrition, Rockville, MD, USA) for maintaining adult rats [24]. The increase in the levels of Trp in the casein amino acid profile was adapted (RHOSTER, São Paulo, Brazil) based on studies previously reported [15,16,17]. Rats receiving a diet supplemented with Trp had 7.6 g of Trp/kg, whereas those receiving a control diet had 2.5 g of Trp/kg.

### 2.4. Exercise Training Protocol 

The maximum running speed (MRS) was determined using a test on a treadmill (AVS, São Paulo-SP, Brazil), as described elsewhere [25]. The MRS reached by the rats was considered 100% to calculate the running intensity in training. The low-intensity continuous aerobic exercise protocol (Modified, Sharma et al. [26]) was performed five times/wk for 21 days. In week one, the rats exercised at the speed of 10 m/min (50% MRS) for 30 min. During week 2, the rats exercised at 11 m/min (55% MRS) for min. During week 3, the rats exercise at 12 m/min (60% MRS), for 45 min.

### 2.5. Mechanical Hypersensitivity Test

Mechanical hypersensitivity was determined using a von Frey filament (Insight, Ribeirao Preto—SP, Brazil) as described by others [20,23]. In summary, after 30 min of adaptation to the equipment, hypersensitivity was assessed in the contralateral (i.e., left) hind paw. The stimulus was imposed 3 times with an interval of 30 s. The average value of 3 measurements was considered the threshold of the paw’s withdrawal (g) and adopted as our mechanical hypersensitivity index. Hypersensitivity was assessed before (baseline) and 1, 7 and 21 days after the injections. These evaluations were carried out in an appropriate room (i.e., silent and controlled temperature), on the same weekday (i.e., Wednesdays), and on time (i.e., 8 to 10 a.m.) by one unique evaluator blind to the treatments.

### 2.6. Sample Collection

Two days after the final exercise training session, the rats were killed in an appropriate room (i.e., clean and quiet). Each group was euthanized on different days of the week, between 8 and 10 a.m. After decapitation, a sample of blood was harvested and centrifugalized at 704 g for 10 min. The serum was separated for further analyses of substance P. To analyze the levels of Trp, 5-HT and KYN, the brain was immediately removed and saved at −80 °C. One brain sample was missed in groups C and ASETrp during the harvesting.

### 2.7. Determination of Brain Tryptophan, Serotonin and Kynurenine Levels

Whole brains were homogenized in three volumes of cold methanol containing phenacetin (100 ng/mL) as a control for the extraction procedure (weight per volume), vortexed and centrifugalized at 13,000× *g* for 10 min (4 °C). Activated resin C18 was added to the supernatant (1/5 of the sample weight—10 mg) to remove hydrophobic compounds (fatty acids, remaining proteins). This mixture was then vortexed and centrifugalized again. The buoyant was passed on to another tube, and the solvent was separated using a SpeedVac (Thermo Fisher Scientific, Waltham, MA, USA). The specimens were reconstructed in the same amount of ultrapure water, passed on to polypropylene flasks (100 μL) and deposited in a chilled autosampler (5 °C). The standards of Trp, 5-HT and KYN (96% purity), as well as the other chemicals, were acquired in a Sigma Aldrich (St. Louis, MO, USA) store. We used a Nexera ultra high-performance liquid chromatography (UHPLC) machine (Shimadzu, Kyoto, Japan) coupled with a Shimadzu Shim-Pack XR-ODSIII (C18, 2.2 μm, 80 Å, 2.0 × 150 mm) column, hyphenated to a maXis ETD high-resolution Electrospray ionization quadrupole time-of-flight (ESI-QTOF) mass spectrometer (Bruker, Billerica, MA, USA), and regulated by the Compass 1.7 software package (Bruker) as reported by Danielski et al. [27] for the liquid chromatography coupled with mass spectrometry tests. The KYN/Trp, 5-HT/KYN and 5-HT/Trp ratios were calculated to assess the participation of the kynurenine and serotonin pathways in the brain Trp metabolism [28,29,30].

### 2.8. Substance P Determination

The serum levels of substance P were measured via commercially available ELISA kits (CEA393RA Substance P, Uscn^®^, Wuhan, China), following the manufacturer’s instructions. After the preparatory procedures, the yellow color generated was read at 405 nm (Stat Fax 2100; Awareness Technology, Palm City, FL, USA), and the levels of substance P were expressed as pg/mL.

### 2.9. Statistical Analyses

Data were checked for normality using the Kolmogorov–Smirnov test. The mechanical hypersensitivity data of the experimental groups were compared utilizing two-way repeated measures ANOVA and the post hoc test of Bonferroni. Data for the levels of Trp, 5-HT, KYN and substance P of the experimental groups were compared utilizing one-way ANOVA and Tukey’s post hoc test. All analyses used the SPSS software (IBM SPSS Statistics for Windows, Version 19.0. Armonk, NY: IBM Corp) and *p* values ≤ 0.05 denote statistical significance.

## 3. Results

The contralateral mechanical hypersensitivity was lower in rats injected with acid saline than in control rats at one day post-induction (Table 1). Aerobic exercise reduced the levels of hypersensitivity of the control rats on the 7th and 21st days post-induction. At these time points, supplementation with Trp diminished hypersensitivity, although to a minor degree. Combining the two interventions did not add to the benefit observed with exercise alone.

Table 2 presents the monoisotopic masses of molecular ions and in-source fragments of Trp metabolites.

**Figure 1:**
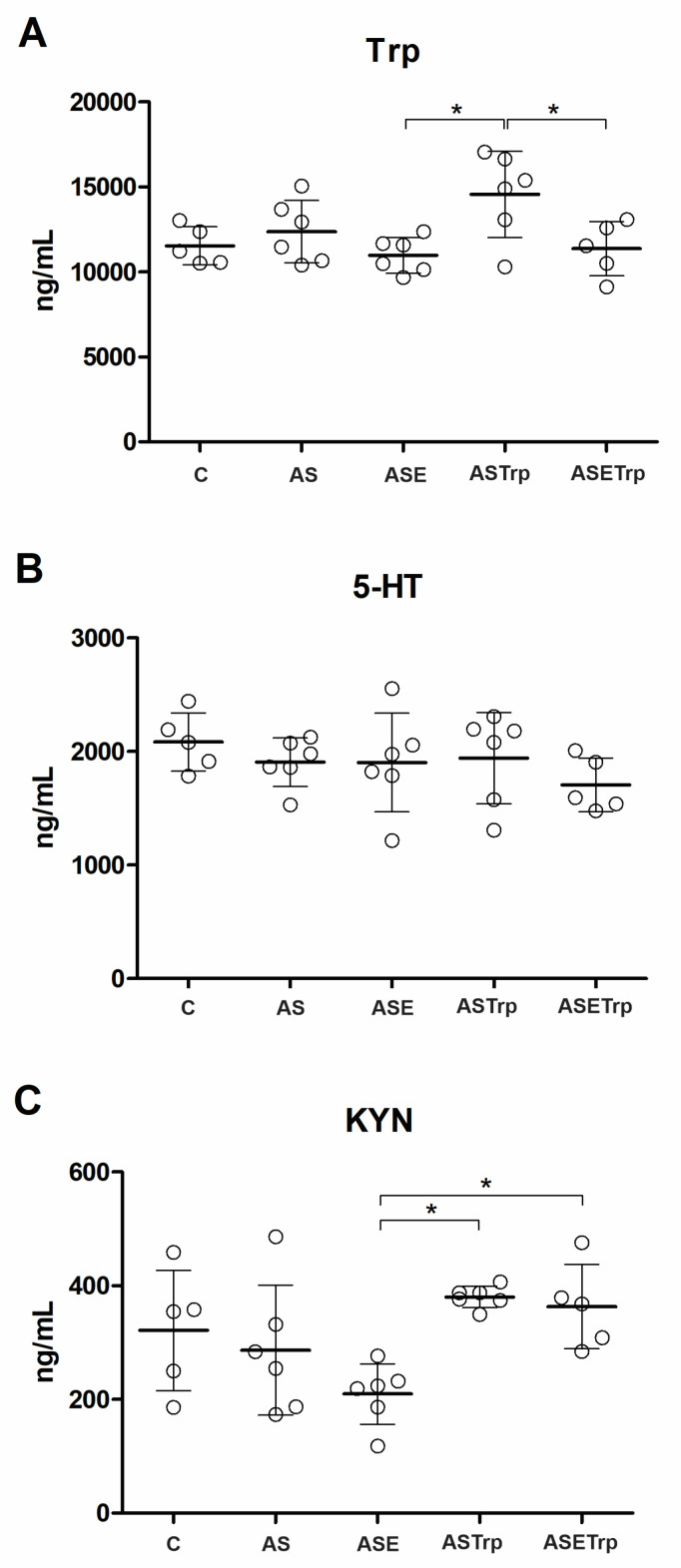
The brain concentrations of tryptophan (**A**) are higher in rats supplemented with tryptophan than in the ASE and ASETrp groups. The brain concentrations of serotonin (**B**) do not differ between groups. The brain concentrations of kynurenine (**C**) are lower in the ASE group than in the ASTrp and ASETrp groups. Trp, tryptophan. 5-HT, serotonin. KYN, kynurenine. C, control; AS, acid saline; ASE, acid saline + exercise; ASETrp, acid saline + exercise + tryptophan; ASTrp, acid saline + tryptophan. Data are means ± SD. * *p* < 0.032.

After 21 days of treatments, the brain levels of Trp, 5-HT and KYN did not alter when comparing control and AS groups (Figure 1). Concerning the impact of interventions, rats from the ASTrp group displayed higher levels of Trp than the ASE and ASETrp groups (Figure 1A). The levels of 5-HT, however, did not alter between groups (Figure 1B). Rats in the ASTrp and ASETrp groups presented higher levels of KYN (Figure 1C) than those in the ASE group.

**Figure 2:**
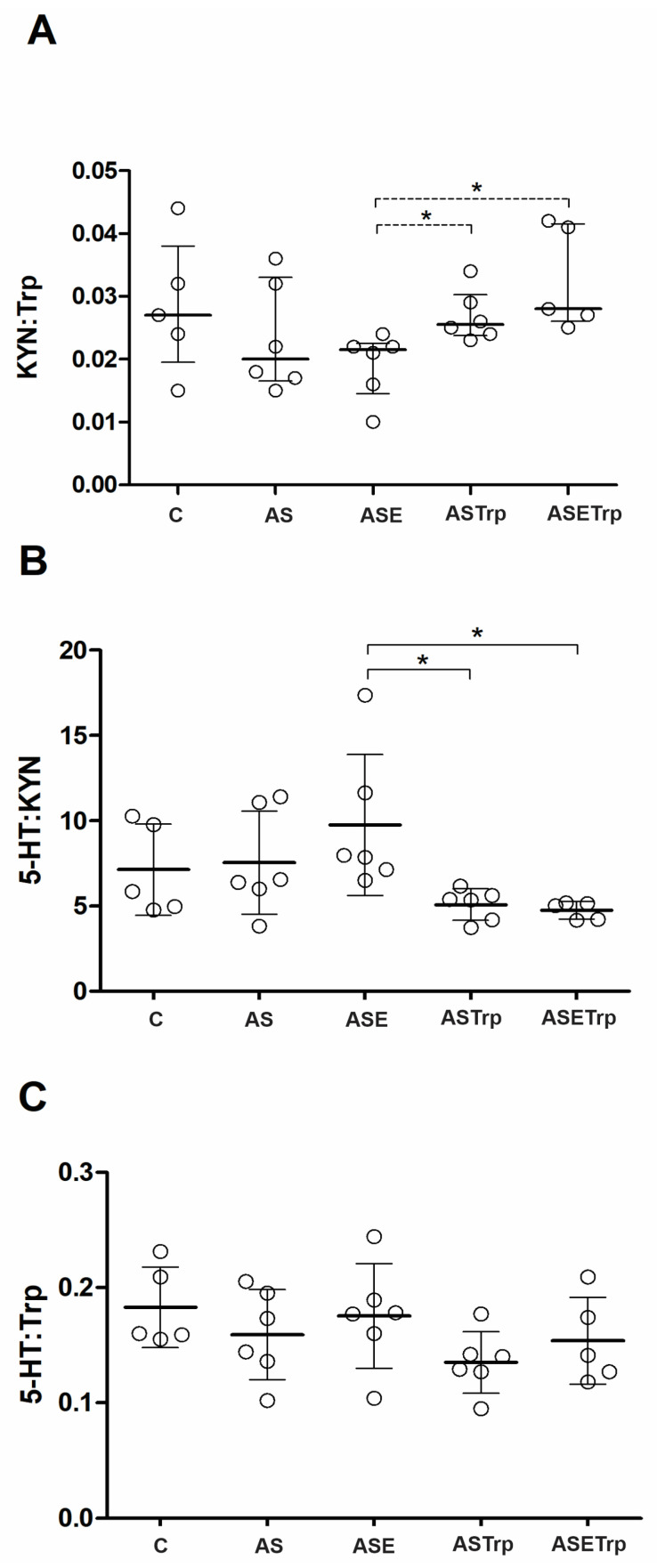
The indolamine 2,3-dioxygenase activity in the brain inferred by the KYN:Trp ratio (**A**) is higher in the ASTrp and ASETrp group than in the ASE group. The brain 5-HT:KYN ratio (**B**) is lower in the ASTrp and ASETrp than in the ASE group. The brain 5-HT:Trp ratio (**C**) is not different between groups. Trp, tryptophan. 5-HT, serotonin. KYN, kynurenine. C, control; AS, acid saline; ASE, acid saline + exercise; ASETrp, acid saline + exercise + tryptophan; ASTrp, acid saline + tryptophan. Data are means ± SD. * *p* < 0.048.

Despite the differences in the brain levels of Trp, curiously, the activity of IDO (i.e., KYN:Trp ratio) (Figure 2A) and the 5-HT:KYN ratio (Figure 2B) did not differ between the ASTrp and ASETrp groups. However, these groups had higher brain KYN concentrations (see Figure 1C), higher inferred IDO activity (Figure 2A) and a lower 5-HT:KYN ratio (Figure 2B) than the ASE group. The 5-HT:Trp ratio was not statistically distinct between groups (Figure 2C).

**Figure 3:**
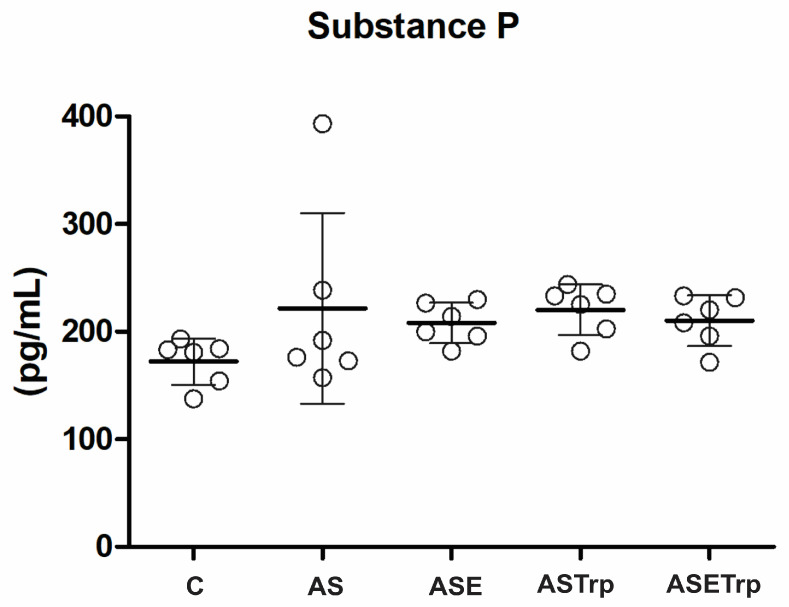
The serum concentrations of substance P are not different between groups. C, control; AS, acid saline; ASE, acid saline + exercise; ASETrp, acid saline + exercise + tryptophan; ASTrp, acid saline + tryptophan. Data are means ± SD.

After the period of interferences, the serum levels of substance P were not statistically different amongst the groups in this experiment (Figure 3).

## 4. Discussion

We submitted female rats injected with acid saline to supplementation with Trp and a low-intensity continuous aerobic exercise, either solely or in conjunction, for 21 days to test its effects on the modulation of mechanical hypersensitivity.

The acid saline model used here caused contralateral mechanical hypersensitivity for around 21 days. Nevertheless, our results demonstrate that the brain levels of Trp, 5-HT, and KYN were not influenced (C = AS). Such findings indicate that mechanical hypersensitivity may not be related to disruptions in the brain metabolism of Trp and hence in its metabolites’ homeostasis in the studied model. Nonetheless, our results are in alignment with the hypothesis that the model for chronic pain induced by acid saline reduces the central concentration of serotonin and augments that of KYN, as reported elsewhere [7,21]. In a previous study [20], however, increased levels of muscle interleukin 6 (IL-6) and serum cortisol in the acid saline model were reported, which suggests that the HPA (hypothalamic–pituitary–adrenal) axis is predominantly affected. Indeed, there is evidence that high serum concentrations of cortisol associated with stress might worsen ache in individuals with fibromyalgia [31] and potentialize the pro-nociceptive influence of inflammatory markers like IL-6 and tumor necrosis factor-alpha (TNF-α) [32,33]. Moreover, here, we noted that the serum levels of substance P were similar in both the control and AS groups. Such results indicate that the serotoninergic modulation system appears not to be affected by acid saline-induced hypersensitivity since substance P concentrations are negatively modulated by efferent serotoninergic neurons [9,34].

Regarding the Trp supplementation effects, it significantly reduced (~67%) the hypersensitivity in the ASTrp group (ASTrp < AS), and in the brain it augmented the levels of Trp in animals that had not exercised (ASTrp > ASE and ASETrp). In this experiment, the ratio of Trp to large neutral amino acid (LNAA) in the diet supplemented with Trp (Trp/LNAA: 15.32) was higher in comparison with that in the control aliment (Trp/LNAA: 5.4). It seems, therefore, that Trp addition favored its influx to the brain.

Despite the increased brain Trp concentrations, the levels of 5-HT in the ASTrp group did not augment, which diverges from other studies [35,36]. This result must be interpreted with caution. The elevation in the brain 5-HT concentration seems to occur in specific regions of pain modulation by the serotoninergic system (i.e., hypothalamus, ventromedial rostral bulb, brain stem magnum raphe nucleus) [37,38]. Thus, the dilution of these brain structures rich in this neurotransmitter by the total brain mass to produce homogenates has masked some differences between groups in this work.

Although the brain Trp concentrations were different among experimental groups (ASTrp > ASE and ASETrp), curiously, the levels of KYN, the activity of IDO and the 5-HT/Trp ratio were not divergent between the ASTrp and ASETrp groups. These groups had higher brain KYN and IDO activity levels and a lower 5-HT/KYN ratio than the ASE group. It is conceivable that mediators of inflammation, like interleukin 1β, IL-6 and TNF-α, that are elevated in the brain of animals with acid saline-induced fibromyalgia [39] may activate IDO [40], which, in the presence of an excess of Trp, would lead to increased KYN levels in the brain. This hypothesis is supported by the literature since around 95% of Trp in the diet is metabolized via the KYN pathway. In comparison, only 1–2% are processed via the serotonin pathway [41].

Concerning the aerobic exercise employed here, our results show its efficacy in restoring the hypersensitivity to the degrees of control animals. Regular aerobic exercise is suggested to augment the plasma levels of free Trp (Trp-F). In contrast, the levels of LNAA are diminished because of its elevated muscular absorption and metabolism during exercise [42]. Therefore, the ratio Trp-F/LNAA increases, which results in elevated Trp-F influx towards the brain, leading to an increase in the levels of serotonin in the brain [43,44]. Endogenous opioids and serotonin are suggested to play important roles in analgesia produced by exercise in humans and animals [45,46]. However, we found no increase in the brain levels of Trp and 5-HT in animals with hypersensitivity who underwent the exercise program. It is conceivable that the restored hypersensitivity demonstrated here is linked to the opposed influence of the applied exercise program to inflammation (i.e., reduced muscle IL-6) and to stress (i.e., reduced cortisol) [14,20], which indicates normalization of the HPA axis activity.

The association of interventions, nevertheless, did not diminish hypersensitivity notably (~11%), in comparison with exercise alone (ASE = ASETrp). In addition, the brain levels of Trp and 5-HT were not influenced by the combined interventions. Animals from the ASETrp group had probably used the excess of Trp given by supplementation as an energy source to exercise by converting Trp into acetyl-CoA via Trp metabolism (i.e., Kegg:map00380). Acetyl-CoA is fuel to the Krebs cycle and, hence, oxidative phosphorylation. Thus, less Trp would reach these animals’ brains.

Finally, this study has limitations. First, we did not determine the levels of 3-hydroxykykynurenine, which could indicate the possible neuroprotective effect of the treatments in the model using the kynurenic acid to 3-hydroxykykynurenine ratio. Moreover, the acid quinolinic acid levels, which are important in the KYN pathway, were not measured. Such analyses would help to have a clear window into the fibromyalgia-like model in female rats. And second, we did not evaluate spontaneous behavior like locomotor activity in the animals. Such measurement would add important information about the effects of the treatments on the used model. Therefore, we encourage the inclusion of assessments of these parameters in future studies.

## 5. Conclusions

In conclusion, mechanical hypersensitivity in the studied fibromyalgia-like model in female rats appears to be modulated by Trp supplementation because of increased brain IDO activity that leads to improved Trp metabolism via the KYN pathway. However, mechanical hypersensitivity is not modulated by low-intensity continuous aerobic exercise through brain Trp metabolism via the KYN or serotonin pathways. Finally, the conjunction of interventions does not have positive synergic actions in modulating mechanical hypersensitivity in female rats. Because this is a short study, generalizing its findings warrants caution.

## Figures and Tables

**Table 1:** Mechanical hypersensitivity in the contralateral paw measured by von Frey filaments.

	Baseline	1 Day Post-Injection	7 Days Post-Injection	21 Days Post-Injection
C (g)	36.69 ± 0.76	36.44 ± 2.45	35.24 ± 0.33	36.11 ± 1.38
AS (g)	35.95 ± 0.84	17.14 ± 1.36 ^#^	14.36 ± 1.39 ^#^	14.92 ± 2.09 ^#^
ASE (g)	37.09 ± 0.45	13.42 ± 1.49 ^#^	38.01 ± 0.79 *	36.54 ± 0.99 *
ASTrp (g)	36.98 ± 0.35	12.81 ± 1.72 ^#^	18.49 ± 3.99 *^#^	21.89 ± 3.69 *^#^
ASETrp (g)	36.32 ± 0.46	11.64 ± 1.27 ^#^	36.96 ± 0.41 *	38.15 ± 1.82 *

Data are means ± SD; C, control; AS, acid saline; ASE, acid saline + exercise; ASTrp, acid saline + Trp supplementation; ASETrp, acid saline + exercise and Trp supplementation. # *p* < 0.05 vs. C group. * *p* < 0.05 vs. AS group.

**Table 2:** Monoisotopic masses of molecular ions and in-source fragments of Trp metabolites.

Metabolite	*m*/*z* [M + H]^+^	*m*/*z* In-Source Fragments
Tryptophan	205.0972	188.071 (100)^1^
Kynurenine	209.0920	192.066 (37). 136.076 (15). 94.065 (8)
Serotonin	177.1022	160.076 (280)

## Data Availability

The data will be shared on reasonable request to the corresponding author.
